# The absence of alternative stable states in vegetation cover of northeastern India

**DOI:** 10.1098/rsos.211778

**Published:** 2022-06-15

**Authors:** Bidyut Sarania, Vishwesha Guttal, Krishnapriya Tamma

**Affiliations:** ^1^ Centre for Ecological Sciences, Indian Institute of Science, Bengaluru 560012, India; ^2^ School of Arts and Sciences, Azim Premji University, Bengaluru 562125, India

**Keywords:** spatial ecology, remotely sensed data, state diagram, northeast India

## Abstract

Globally, forests and savannah are shown to be alternative stable states for intermediate rainfall regimes. This has implications for how these ecosystems respond to changing rainfall conditions. However, we know little about the occurrence of alternative stable states in forest ecosystems in India. In this study, we investigate the possibility of alternative stable states in the vegetation cover of northeastern India, which is a part of the Eastern Himalaya and the Indo-Burma biodiversity hotspots. To do so, we construct the so-called state diagram, by plotting frequency distributions of vegetation cover as a function of mean annual precipitation (MAP). We use remotely sensed satellite data of the enhanced vegetation index (EVI) as a proxy for vegetation cover (at 1 km resolution). We find that EVI exhibits unimodal distribution across a wide range of MAP. Specifically, EVI increases monotonically in the range 1000–2000 mm of MAP, after which it plateaus. This range of MAP corresponds to the vegetation transitional zone (1200–3700 m), whereas MAP greater than 2000 mm covers the larger extent of the tropical forest (less than or equal to 1200 m) of northeast India. In other words, we find no evidence for alternative stable states in vegetation cover or forest states at coarser scales in northeast India.

## 1. Introduction

Ecosystems are complex dynamical systems comprising various interacting, strongly interdependent biotic and abiotic components, with both positive and negative feedbacks between them [1,2]. The existence of features such as feedback loops and stochasticity—both environmental and demographic—can lead to interesting relationships between the state of the ecosystems and their drivers. Most generally, ecosystems may exhibit linear or nonlinear relationships between ecosystem state and the environmental drivers. In systems with strong positive feedback, ecosystems may exhibit multiple stable states, i.e. for a given driver value, an ecosystem can be present in contrasting states that depend on the historical conditions [1]. Empirical evidence for the existence of such alternative stable states has mounted over the past two decades, ranging from grasslands and various forest types to lakes to corals [3–10].

The nature of the relationship between the ecosystem state and the environmental driver has important consequences for how ecosystems respond to changing driver variables. Consider a system with a linear relationship between the state variable and the driver; such a system will probably respond to the changing environmental driver gradually and predictably [1]. By contrast, a system with a nonlinear relationship can show rapid changes in response to changing driver conditions. On the other hand, ecological systems with alternative stable states (referred to as multi-stable systems) are characterized by two key features: abrupt transitions and hysteresis. In multi-stable systems, when the driver crosses a threshold value, a rapid transition from one stable state to an alternative state may occur [1]. Such transitions, also called regime shifts, can also occur due to stochasticity [8,11,12]. These transitions can dramatically alter the structure and functioning of these ecosystems. Furthermore, ecosystem states are difficult to reverse to the previous state, due to the phenomenon of hysteresis [1,13]. Therefore, it is important to characterize the relationship between key environmental drivers and ecosystem states. This will enable us to project how ecosystems may respond to changing environmental conditions, including those caused by climate change.

In the context of forest and savannah ecosystems, it is now well established that mean annual precipitation (MAP) is an important driver of tree cover, globally, at large spatial scales [4,5,7,14]. Intriguingly, tree cover in Africa (and parts of Australia and South America) shows alternative stable states, with potential for abrupt transitions and hysteresis [5]. However, the relationship between vegetation cover and MAP is complex and is not similar across continents [15]. As climate change and other human interventions threaten the forest and savannah ecosystems, characterizations of relationships between ecosystems and their key drivers—via the so-called state diagrams (see box 1)—are important [16].

Box 1. **State diagrams.**

To understand how ecosystems respond to a changing driver, we construct the so-called state diagram. This is a plot of how the state variable of ecosystem varies as a function of the environmental driver [4,7], using the mode of the state variable rather than the mean. To illustrate what such a diagram can reveal, we consider two possibilities: (i) a uni-stable system, where the ecosystem shows only one stable state as a function of the driver; here, the ecosystem responds gradually in response to changing driver values (box figure *a*); (ii) a bi-stable system, where the ecosystem exhibits two alternative stable states for an intermediate range of driver values; in such scenarios, the ecosystem can abruptly switch between the two states (box figure *b*).

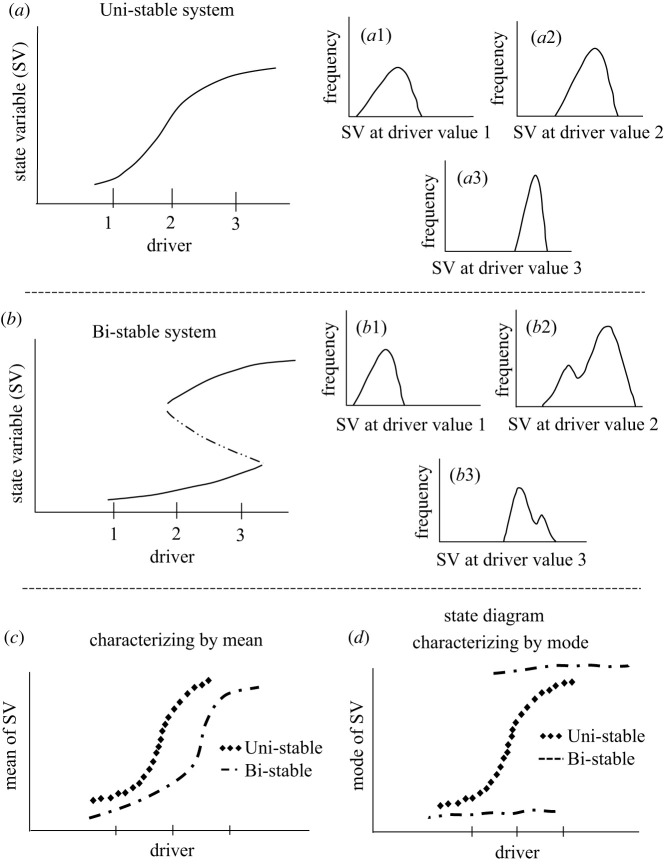

**Box Figure.** Contrasting features of uni-stable and bi-stable states, and the idea of state diagrams: SV in the figure stands for state variable. Three representative driver values are marked 1, 2 and 3 on the *x*-axis of figure *a*,*b*; (*a*) shows a uni-stable system, with figure *a*1–*a*3 showing how frequency distributions of the observed state variable (SV) change with driver values. Note that the frequency distributions are unimodal for all values of the driver; (*b*) shows a bi-stable system, where the state variable can take values corresponding to two stable states for an intermediate range of driver values. The corresponding figure *b*1–*b*3 shows that the frequency distributions of the state variable will be bimodal when the driver falls in the bi-stable region (figure *b*2 and *b*3); (*c*) shows that the relationship between mean state variable and driver will be qualitative similar for both uni-stable and bi-stable systems; (*d*) shows that the relationship between mode and the driver—also called the state diagram—will be qualitatively different for uni-stable and bi-stable systems.

Our main focus is to distinguish the above two scenarios in empirical datasets. Let us say that we have high-quality data available, i.e. we have sufficient number of measurements of the ecosystem state variable for a range of environmental conditions. With such data, we compute the frequency distribution of the state variable for each of the environmental conditions. For scenario (*a*), the uni-stable system, we expect the frequency distribution of the state variable to be unimodal for the entire range of environmental conditions (box figure *a*1–*a*3) [2]. The mode of the distribution corresponds to the stable state of the ecosystem for a given driver value; the mode changes gradually as a function of the environmental driver. By contrast, for scenario (*b*), the bi-stable system, the frequency distribution of the state variable is expected to show two modes in the intermediate driver values, corresponding to two alternative stable states of the ecosystem (box figure *b2*–*b*3) [2].

Consider the standard regression analysis between the state variable and the driver. This is analogous to plotting and analysing mean as a function of the driver. In this analysis, both the uni-stable and bi-stable systems show qualitatively similar features (box figure *c*). Hence, this approach cannot distinguish between uni-stable and bi-stable systems.

By contrast, let us plot the mode of the frequency distribution as a function of driver values—called the state diagram. As shown in box figure *d*, this plot can differentiate whether the system is a uni-stable system or a bi-stable system. Therefore, we have chosen the method of state diagram for our analysis of remotely sensed vegetation cover data from northeastern India.

Despite the presence of a wide variety of climatic zones and forest types in India, no such characterization has been carried out for Indian ecosystems [4,14,17–19]. MAP in India shows strong spatial gradients, which in turn influences the diversity of vegetation types [18]. Tropical and subtropical forests (evergreen, semi-evergreen and moist deciduous) are typically found in areas of high rainfall (greater than 1500 mm), whereas dry deciduous, thorny and scrub forests are typically found in areas of low rainfall (less than 1000 mm) [20]. Interestingly, forest-grassland complexes such as tropical montane forests and grasslands of Western Ghats (shola-grassland mosaics) exist as alternative stable states, with frost maintaining a stable boundary between the two stable states [21]. Apart from this, other vegetation mosaics have also been reported, including woodland-grassland of western India [22], and forest-grasslands/shrub-lands of the Himalayas [23–26]. While the vegetation cover in Africa and South America has been shown to exhibit alternative stable states [4,5,7,14], no studies have examined whether vegetation cover in India shows alternative stable states. In this study, we focus on vegetation in northeast India (henceforth NEI) which is a part of two biodiversity hotspots—the Indo-Malayan and the Eastern Himalaya [27]. The Eastern Himalayas along with the hills of NEI (including the Garo-Khasi-Jaintia hills, Arakan hills) also show tremendous diversity in vegetation and rainfall. Over the past few years, there is evidence of decreasing rainfall and increasing temperature, with reports of unusual drought conditions in many parts of the NEI region [28–30]. These changes have been linked to rising global temperatures, which reduce the transport of moisture over NEI, resulting in less rainfall than normal [31]. Characterizing how vegetation ecosystems in this region respond to key environmental drivers, such as rainfall, is therefore essential for the long-term monitoring and conservation of these systems.

Here, our aim is to investigate whether vegetation cover of NEI show alternative stable states. To do so, we construct the so-called state diagram (see box 1 for more details) for the vegetation cover of NEI. If the NEI ecosystems show alternative stable states, we expect that vegetation cover will exhibit frequency distributions with multiple modes. On the other hand, if the NEI ecosystems show uni-stable state, we expect that vegetation cover will exhibit unimodal frequency distributions.

## 2. Methods

### 2.1. Study site

**Figure 1.**
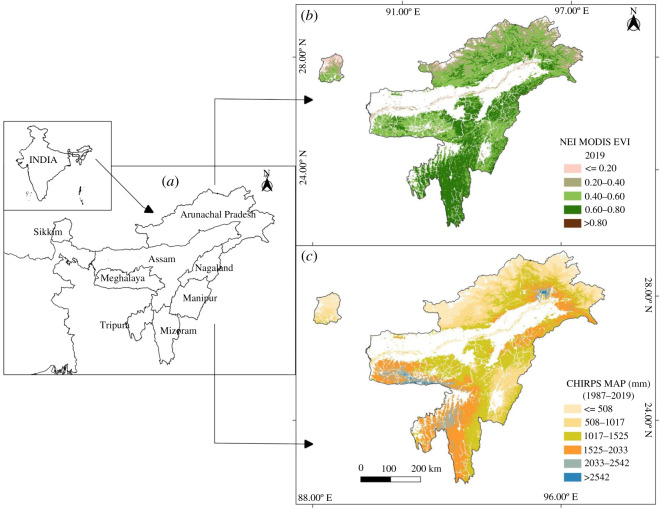
(*a*) Map of NEI showing eight states of the region, (*b*) spatial distribution of EVI, and (*c*) MAP. From both these images (*b* and *c*), pixels with a high human footprint have been removed (see Methods for details).

NEI consists of eight states (Sikkim, Assam, Meghalaya, Arunachal Pradesh, Nagaland, Mizoram, Tripura and Manipur) and comprises almost 8% of India's total landmass (figure 1). The states of Sikkim, Arunachal Pradesh and the northernmost part of Assam belong to the eastern Himalaya biogeographic realm, while the southernmost parts of Assam and the states of Meghalaya, Mizoram, Tripura, Manipur and Nagaland belong to the Indo-Malayan biogeographic realm.

NEI region encompasses a wide elevational range from 20 m to approximately 7000 m above mean sea level, resulting in a large climate gradient that ranges from tropical, subtropical, temperate to alpine [32]. The NEI region receives the highest rainfall during the southwest monsoon of the Indian subcontinent, but the amount of rainfall at local scales is highly variable due to the rain shadow effects [32,33]. The steep elevational gradient, along with the heterogeneity in rainfall across NEI, results in various vegetation types, including tropical, subtropical and temperate forests, grasslands (both cold-arid and flooded) and savannah [26,34–36].

### 2.2. Vegetation cover data

Following recent works to investigate alternative stable states of tree cover [4,5,7,14], we considered vegetation cover as the state variable and MAP as the driver. We used the enhanced vegetation index (EVI), obtained from the Moderate Resolution Imaging Spectroradiometer (MODIS), as a proxy for the vegetation cover. EVI is an atmospherically corrected index, which is less sensitive to soil background, and more sensitive to canopy variation [37]. Thus, EVI is well suited for tropical regions with high biomass compared with other vegetation indices such as normalized difference vegetation index (NDVI) and vegetation continuous fields (VCF) [38]. We obtained EVI at 1 km resolution obtained from MODIS (dataset: MCD43A4), using the Google Earth Engine platform [38,39]. We constructed a composite based on images obtained in the post-monsoon season (1 October–30 November 2019). This period was chosen as the vegetation cover reflects the highest possible in the growing season, while minimizing cloud cover.

### 2.3. Rainfall and elevation data

We used rainfall data for the period spanning 1989–2019 from the Climate Hazards Group InfraRed Precipitation dataset [40]. We calculated the mean of the annual precipitation (MAP) by averaging over the 30-year window and downloaded the data at a spatial resolution of 1 km. We obtained elevation data from the Shuttle Radar Topography Mission (SRTM) data at 1 km resolution [41].

We used global terrestrial human footprint maps (HFI) and GlobCover maps as masks to eliminate pixels with high human footprint, especially those associated with built-up and agricultural areas [7,42,43]. The HFI value of NEI ranges from 0 to 43, and typically most pixels associated with natural vegetation lie within the range of 0 to 8 (See electronic supplementary material, S1.A). Thus, we removed pixels with HFI values greater than 8 from our analysis. We also analysed the data without removing the human footprint (see electronic supplementary material, S1.B). Additionally, we considered a few other possible drivers such as temperature, slope and aspect; since these are not the focus of our paper and nor do they affect the primary conclusions, the corresponding methods and results are reported in the electronic supplementary material, S1.D.

We processed and analysed all datasets via the Google Earth Engine platform (for code link see electronic supplementary material, S2).

### 2.4. State diagram

A state diagram is a plot of the mode of the frequency distribution of vegetation cover as a function of the MAP (see box 1 for details). We broadly followed the methods of Majumder *et al*. [7] to construct the state diagram. First, we aggregated the MAP into 100 mm bins. For each precipitation bin, we identified all pixels and obtained the corresponding EVI data. We then constructed a smoothed frequency distribution of the EVI using the function ‘density’ in R. From this, we identified the modes (local maxima) of the distribution. A local maxima is considered as a distinct mode if it satisfies two conditions: (i) the ratio between two local maxima is greater than 0.25, and (ii) the distance between the two local maxima is greater than 0.1 EVI units. If the ratio is greater than 0.25 but the distance between the mode is less than or equal to 0.1, then the weighted average of modes is taken. This approach has been adopted to avoid small peaks that appear due to unknown stochastic reasons [7]. The sorted EVI modes for each precipitation bin are then plotted as a function of MAP.

To determine the influence of elevation on vegetation patterns, we also constructed a state diagram of EVI with elevation as the driver. Following the same protocol (described above), we divided elevation into 100 m bins and obtained the modes of EVI for each bin.

## 3. Results

**Figure 2.**
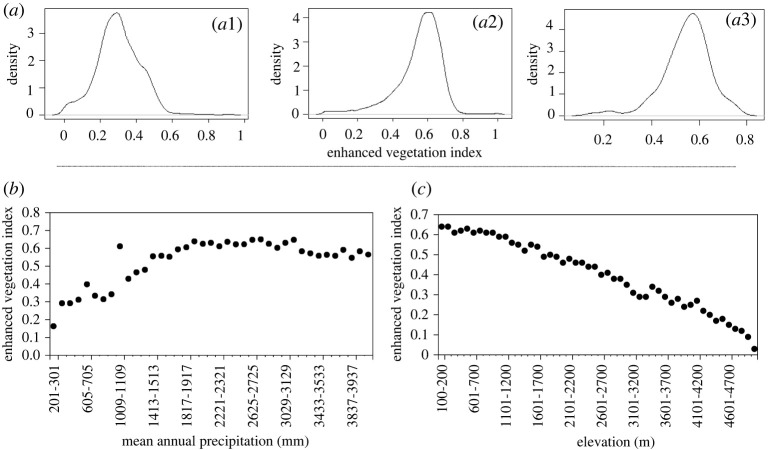
Frequency distribution of EVI (smoothed showing density function in R) for different rainfall bins corresponding to (*a*1) 403–503 mm, (*a*2) 1009–1109 mm and (*a*3) 4039–4139 mm. The observed distributions are all unimodal. (*b*) State diagram, where mode of EVI increased gradually with MAP. (*c*) Mode of EVI decreased monotonically with an increase in elevation. The rainfall bins are 100 mm bins, while the elevation bins are 100 m bins.

The state diagram of vegetation cover for the NEI is indicated in figure 2. From this plot, we observed that EVI increases linearly with MAP up to 2000 mm, beyond which there is no further increase in EVI. We did not find evidence for bimodality in EVI for any rainfall regime (figure 2*a*1–*a*3), which suggests that the system does not show bi-stability (or the presence of alternative stable states). We also investigated drivers other than MAP. We found that EVI decreased monotonously with the increase in elevation (figure 2*c*), again showing unimodal distribution for the entire range of elevation.

**Figure 3.**
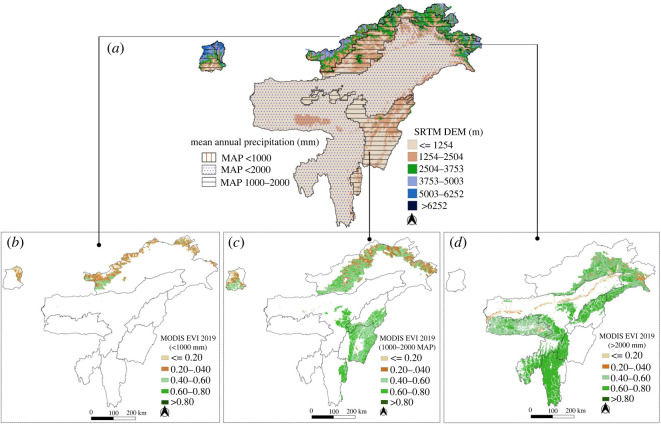
(*a*) Elevation, and (*b*) spatial distribution of EVI for MAP of less than or equal to 1000 mm, (*c*) 1000–2000 mm and (*d*) greater than 2000 mm. Elevation gradients map shows that MAP of 1000 mm corresponds to greater than 2500 m, 1000–2000 mm corresponds to greater than 1200–3700 m elevation and greater than 2000 mm MAP corresponds to less than 1200 m elevation.

To investigate this relationship further, we examined the relationship of three large bands of precipitation with elevation and vegetation cover (figure 3). The three bands were low (less than or equal to 1000 mm), intermediate (1000–2000 mm) and high (greater than or equal to 2000 mm), as these bands had a distinct response of frequency distributions of EVI as a function of MAP in the state diagram. Regions with low MAP (less than or equal to 1000 mm) showed higher elevation profile (greater than or equal to 2500 m; figure 3*a*,*b*), while regions with a higher range of MAP (greater than or equal to 2000 mm) typically showed low elevation profile (less than or equal to 1000 m; figure 3*a*,*d*). On the other hand, the regions with intermediate range of MAP (1000–2000 mm) include elevational gradients that span a wider range of elevations (greater than 1000–3700 m; figure 3*a*,*c*). These observations are consistent with the decreasing trend of EVI with increasing elevation.

## 4. Discussion

In this study, we constructed a state diagram for vegetation cover, by characterizing the mode of frequency distributions of EVI as a function of MAP for NEI. The state diagram shows that the EVI gradually increased with MAP and reached an asymptote at values greater than 2000 mm. Throughout this range of rainfall values, we did not find evidence for bimodal distribution of vegetation cover, indicating an absence of bi-stable forest/savannah states in NEI at these large scales. We also found that EVI monotonically decreases as elevation increases; furthermore, higher elevations are associated with reduced MAP and lower temperatures, both factors possibly contributing to lower EVI. This is consistent with other studies showing a decrease in tree species richness as elevation increases [35,44]. Our study provides the first assessment of the large-scale relationship between vegetation cover and rainfall for northeastern India in the context of forest resilience and multiple stable states.

### 4.1. The absence of alternative stable states

We note the contrast with the characteristics of coarse-scale vegetation in Africa, South America and some parts of Australia, which exhibit bimodal distribution of tree cover at intermediate mean annual rainfall regimes corresponding to 1000–2500 mm [4,5,7,14]. Although there is no pattern of bimodality observed for NEI, there is a monotonic increase in vegetation cover (EVI) at intermediate rainfall regimes (1000–2000 mm of MAP). This intermediate rainfall regime corresponds to a vegetation transitional zone (1200–3700 m elevation) of NEI, where we find a variety of forests: subtropical broadleaved forest at elevations greater than 1200–2000 m, mixed coniferous forest or temperate forest at greater than 2000–3000 m and sub-alpine vegetation types at elevations of greater than 2900 m [36]. As a result, the monotonic increase in EVI at intermediate MAP could be due to the presence of different vegetation types. On the other hand, at greater than 2000 mm MAP, typically tropical evergreen and semi-evergreen forests are dominant, which explains the plateau observed in the state diagram [34].

Although we did not find bimodality in NEI at the coarse scale (1 km), we do not rule out that small patches of forest-grassland mosaics occur. Indeed, several small pockets of grassland and savannah are found in the hills and valleys of NEI [26,45], including Dzuku valley, Balpakram NP, Kaziranga NP and Manas NP, apart from the high-elevation grasslands [26,45,46]. The natural grassland and savannah ecosystems in NEI occupy a smaller extent compared with the forest ecosystems. In addition, the savannah and grasslands of the Brahmaputra valley have been altered extensively for agricultural activity. This has further reduced the extent of these ecosystems in the landscape. The presence of forest-grassland mosaics, albeit at very small spatial scales, suggests the presence of mechanisms that maintain such mosaics. Furthermore, landscape fragmentation and resulting enhanced demographic noise can also induce bi-stability and abrupt transitions [12]. By contrast, spatial processes such as dispersal and spatial gradients of environmental driver may increase the resilience of ecosystems and render the transitions more gradual [47]. We argue that fine-scale analysis with high-resolution data is necessary to understand the dynamics and stability of such mosaic landscapes [48].

### 4.2. Drivers beyond rainfall

Vegetation cover is often maintained by a complex interplay of fire, herbivores and dispersal [49–51]. In this study, we aimed to construct a state diagram—as it is ideal for understanding the stability structures of ecosystems. We considered the MAP as a potential driver for ecosystem stability, as previous studies across the continent have shown a strong influence of precipitation in ecosystem stability [4–7,14]. However, this does not rule out the importance of other potential drivers such as fire, altitude, elevation, temperature, aspect and slope in the northeastern ecosystem. Our preliminary regression analysis shows that indeed temperature, elevation and slope have a relation with the state of the ecosystem i.e. vegetation cover (EVI) of NEI (electronic supplementary material, S1.D). However, it is also important to note that, while regression-based analysis can find the relationship between ecosystem state and its drivers, it's insufficient to detect ecosystem bi-stability, as illustrated in box figure *c*,*d*. Future studies can delve into this further, including how we can integrate the state diagram approach with the traditional classical regression analysis for the drivers.

Among many possible drivers, fires are important drivers in many vegetation ecosystems. For instance, as the amount of grass biomass increases, fires become more intense, limiting forest growth, whereas decreases in grass biomass result in less fire that allows the forest to expand. Besides that, herbivores play an important role in the fire–grass interaction, as the degree of grazing affects the load of grass biomass. Slash and burn cultivation (shifting cultivation) is the major reason for forest fire in the NEI; however, the effect of fire and herbivores on the forest's stability remains unclear [26]. Flood, on the other hand, is known to have positive feedback in maintaining floodplain grassland ecosystems of NEI, such as Assam's Kaziranga NP [52]. There is limited knowledge on the interplay of fire, grazing and flood in maintaining forest savannah ecosystems in NEI, which therefore needs further attention.

### 4.3. Response to climate change

Vegetation cover in the NEI region has responded to past changes in climate. Palaeoecological investigations of the recent past—up to 1200 years before present—show that the climate of NEI has been relatively stable [53], possibly, allowing for stability in vegetation structure and composition. However, over longer time frames, such as that of the Holocene, the climate in NEI has probably fluctuated between cool, dry and warm, moist conditions [54]. Such past climate fluctuations have probably driven changes in vegetation. For instance, palaeoecological analyses from Assam provide evidence for shifts between savannah and tropical mixed deciduous forests over the past 10 000 years, including instances of floods in the valley [55,56]. Changes in vegetation patterns in response to changes in precipitation have also been reported from other regions of Eastern Himalayas [57], Garo hills of Meghalaya [58] and from the Arakan range of Nagaland [59]. Therefore, a characterization of the relationship between vegetation cover and rainfall along with palaeological information is important for predicting future changes in vegetation pattern.

Indeed, ongoing climate change in the Himalayas is causing changes in precipitation patterns, seasonality and temperature [60]. The NEI region is already experiencing rainfall deficit and is expected to face drought as a result of future climate change [61]. Vegetation responses to these changes are likely to be complex and spatially heterogeneous [62,63]. Although data on the response of vegetation to the ongoing climate change is sparse from the Eastern Himalayas, palaeological data, along with ongoing ecological studies demonstrate how ongoing and future climate changes in the region can lead to shifts in vegetation, biodiversity loss and loss of ecosystem services in the region [64]. Therefore, a further decrease of MAP (less than 1000 mm), an increase in the incidence of forest fires, and deforestation can lead to shifts in vegetation (such as forest to savannah) or change in the plant community structure. Such changes have indeed been observed in other tropical regions, for instance, in the savannah of Africa [49], and Amazonian rain forests [65]. Therefore, further studies exploring the impact of fires, changing rainfall patterns and deforestation on the NEI forests are needed.

## 5. Concluding remarks

In summary, our study provides a first coarse-scale characterization of how vegetation changes with MAP. Our work may aid a better understanding of vulnerability of ecosystems in the Eastern Himalayas, in the context of large-scale climate change as well as local anthropogenic drivers, especially in regions receiving 1000–2000 mm of MAP. While our study has focused on NEI, there is a need for such an analysis at the scale of the Indian subcontinent. The region has a diversity of ecosystems including savannah, scrub and tropical forests. Such an investigation will provide us with more insights into the structuring of vegetation in the subcontinent.

## Data Availability

The GEE code can be accessed directly via the link provided in the electronic supplementary material, S2 A. R code can be accessed via the GitHub (https://github.com/SabihaMajumder/Inferring-critical-thresholds) also link supplied in the electronic supplementary material, S2 B. Additionally, data and relevant code for this research work are available in GitHub: (https://github.com/Bidyut-haidang/Absence-of-alternative-stable-states) and have been archived within the Zenodo repository (https://zenodo.org/record/6487371#.YmfM_tpBzIU). The data are provided in the electronic supplementary material [66].
